# Prognostic implication of hepatoduodenal ligament lymph nodes in gastric cancer

**DOI:** 10.1097/MD.0000000000006464

**Published:** 2017-03-31

**Authors:** Sung Eun Oh, Min-Gew Choi, Jun Ho Lee, Tae Sung Sohn, Jae Moon Bae, Sung Kim

**Affiliations:** Department of Surgery, Samsung Medical Center, Sungkyunkwan University School of Medicine, Gangnam-gu, Seoul, Korea.

**Keywords:** 12a lymph node, D2 dissection, gastrectomy, gastric cancer, hepatoduodenal lymph node, prognosis, stage

## Abstract

There has been controversy regarding whether hepatoduodenal lymph node (HDLN) metastasis in gastric cancer is distant or regional metastasis. HDLN positivity was classified as distant metastasis in the 7th American Joint Committee on Cancer (AJCC) classification, but it was reclassified as regional lymph node metastasis in the 8th AJCC classification. The aim of our study is to verify prognostic significance of HDLN metastasis in gastric cancer.

This retrospective study enrolled patients with gastric cancer who underwent D2 gastrectomy from January 2007 to June 2010. HDLN was classified as a regional lymph node.

Total number of patients was 3175; 143 (4.5%) of them had HDLN metastasis. The HDLN positivity was significantly associated with older age, more advanced tumor stage, undifferentiated histologic type, and pathologic diagnosis of lymphatic, vascular, and perineural invasions. Five-year survival rate of HDLN-positive patients with stages I to III disease was significantly higher than that of stage IV group (59.3% vs 18.8%, *P* = 0.001). In patients with stage III disease, 5-year survival rate of HDLN-positive group was significantly lower than that of HDLN-negative group (51.7% vs 66.3%, *P* = 0.001). Multivariate analysis showed that HDLN metastasis was an independent prognostic factor.

HDLN has a different prognostic significance from other regional lymph nodes in advanced stage of gastric cancer though its positivity is not considered as distant metastasis. HDLN positivity itself seems to be an independent prognostic factor in gastric cancer, and the survival outcomes of patients with stage III disease need to be reconsidered according to HDLN positivity.

## 1 Introduction

The standard surgical treatment for resectable gastric cancer is radical gastrectomy with regional lymph node (LN) dissection.^[1,2]^ There has been controversy regarding the outcome of D2 dissection in patients with gastric cancer because of the higher morbidity and mortality rate after D2 dissection in Western countries.^[3]^ However, recent studies have shown that D2 dissection can be safely performed in experienced centers^[4–6]^ and might be a better choice in patients with advanced gastric cancer with LN metastasis.^[7,8]^ Gastric cancer surgeons in Korea usually remove the hepatoduodenal lymph nodes (HDLNs), especially the No. 12a LNs (the LNs along the proper hepatic artery), during D2 dissection because they are considered regional LNs.

However, controversy has existed over whether HDLN metastasis should be treated as regional or distant metastasis. The 7th American Joint Committee on Cancer (AJCC) staging classified HDLN positivity as distant metastasis.^[9,10]^ In contrast, the Japanese Gastric Cancer Association considered the HDLN as one of the regional LNs.^[11]^ Although the 7th AJCC classification was known to provide more accurate discrimination of patient survival or disease-free survival than the 6th edition,^[12–14]^ some critics had proposed that it was inappropriate to classify HDLN metastasis as distant metastasis.^[15,16]^ A previous study, which was done in Korea, enrolled 1872 patients with gastric cancer having gastrectomy and D2 dissection with removal and pathological assessment of HDLN. This study demonstrated that the survival of patients with stages I to III disease with HDLN metastasis was better than that of patients with stage IV disease without HDLN metastasis. It also concluded that HDLN should be regarded as one of the regional LNs because there was no significant survival difference between patients with stages I to III disease with HDLN metastasis and those without HDLN metastasis.^[15]^ In this regard, the classification of node grouping has been recently changed in the 8th AJCC criteria, and HDLN is considered as one of the regional LNs.^[17]^

One of the problems with the previous studies is the small number of patients with a long inclusion period. In the present study, we retrospectively reviewed a relatively large number of cases from a single center with the aim of evaluating the impact of HDLN metastasis on the 5-year survival rate of patients who underwent radical gastrectomy.

## 2 Methods

Patients with gastric cancer who had undergone curative or palliative surgery at the Department of Surgery, Samsung Medical Center between January 2007 and June 2010 were analyzed retrospectively. Those who were diagnosed with another malignancy or received neoadjuvant treatment were excluded. In this study, the patients underwent D2 lymphadenectomy and either subtotal gastrectomy with Billroth I/II reconstruction or total gastrectomy with Roux-en Y esophagojejunostomy. And they were divided according to presence of HDLN metastasis and pathologic stage for analysis. Adjuvant chemotherapy was usually recommended after surgery except for patients with stage T1N0 or T2N0 cancers. Patients underwent a follow-up, which was done by telephone calls or outpatient visits, and survival data were obtained from patients’ medical records and the Korean cancer registry.

Twelve clinicopathologic factors such as age, sex, extent of resection, histologic types, depth of invasion, LN metastasis, distant metastasis, pathologic stage, lymphatic involvement, venous involvement, perineural involvement, and HDLN positivity were reviewed from medical records and pathology reports in peer-review manner with 2 independent reviewers to reduce selection bias and misclassification or information bias as a result of retrospective aspect.

Histologic type was categorized as differentiated or undifferentiated. Well or moderately differentiated adenocarcinoma was classified as differentiated, whereas poorly differentiated tubular adenocarcinoma, signet ring cell type, and mucinous adenocarcinoma were assigned to the undifferentiated group. The pathologic stage was classified according to the 8th edition of AJCC classification, and HDLN metastasis was considered regional LN involvement. The study protocol was approved by the institutional review board of Samsung Medical Center, Seoul, Korea (SMC 2016-02-056).

### 2.1 Statistical analysis

Differences in clinicopathologic parameters between patients with and without HDLN metastasis were determined by *t* test, χ^2^ test, and Fisher exact test. The 5-year survival rate was calculated using the Kaplan–Meier method, and the log-rank test was used to determine the significance of the 12 clinicopathological variables. Variables with *P* < 0.05 in univariable analysis by Kaplan–Meier method were included in the multivariable analysis. Multivariable analysis was carried out using a Cox proportional hazards model with the backward logistic regression method to identify independent risk factors of patient survival. The hazard ratio and 95% confidence interval were calculated. *P* < 0.05 was considered statistically significant. Statistical analysis was carried out using the statistical software SPSS version 22.0 for Windows (SPSS, Chicago, IL).

## 3 Results

Among 3175 gastric cancer patients, 143 (4.5%) had HDLN metastasis with a mean (±standard deviation) follow-up duration of 54.4 (±31.4) months. There were significant differences in terms of age, extent of resection, histologic types, depth of invasion, LN metastasis, distant metastasis, pathologic stage, and presence of vascular, lymphatic, and perineural involvement between the 2 groups (Table 1). The HDLN positivity was significantly associated with older age, more advanced tumor stage, undifferentiated histologic type, and pathologic diagnosis of lymphatic, vascular, and perineural invasions.

**Table 1:**
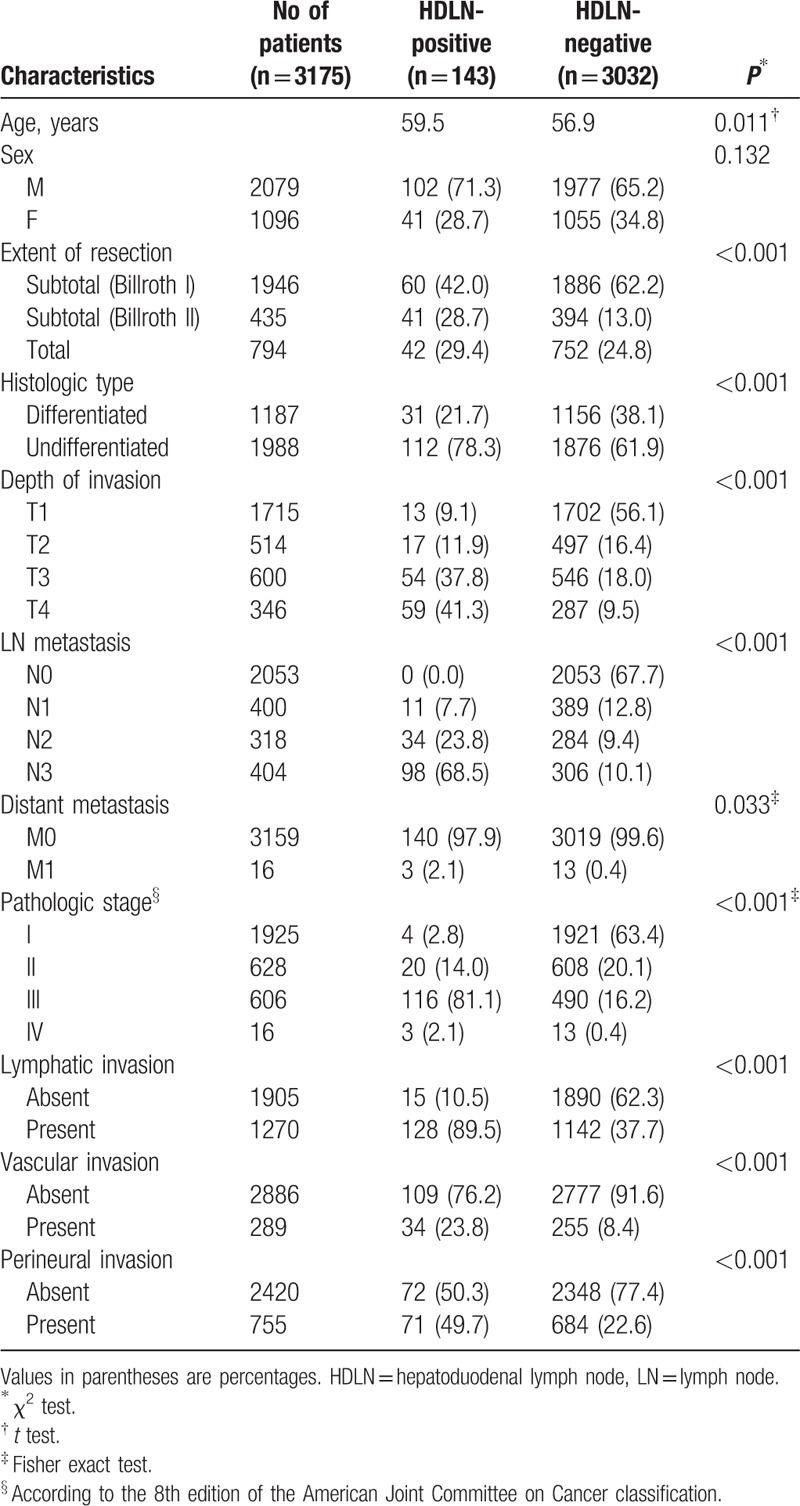
Clinicopathologic characteristics of the HDLN-negative and -positive groups.

**Figure 1:**
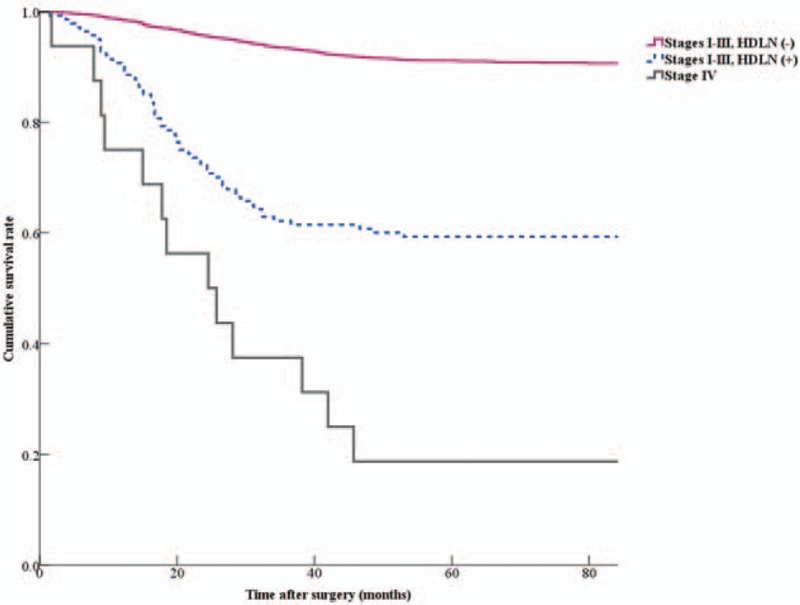
Survival curves of patients with stages I to III disease stratified by status of hepatoduodenal lymph node (HDLN) metastasis and those with stage IV disease. Overall 5-year survival rate of stages I to III disease with HDLN metastasis (59.3%) was significantly shorter than that of stages I to III disease without HDLN metastasis (91.2%, *P* < 0.001) and significantly longer than that of stage IV disease (18.8%, *P* = 0.001).

**Figure 2:**
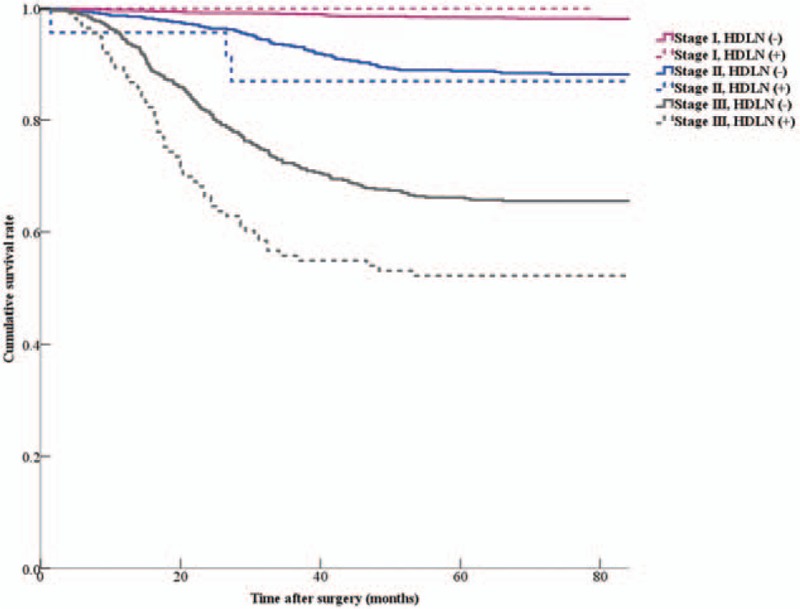
Survival curves of patients with gastric cancer according to stage stratified by status of hepatoduodenal lymph node (HDLN) metastasis. There was a significant difference in 5-year survival rates between the HDLN-positive group (51.7%) and -negative group (66.3%) for patients with stage III disease (*P* = 0.001). There were no significant differences in 5-year survival rates between HDLN-positive and -negative groups for other stages: stage I (100% vs 97.9%, *P* = 0.789), and stage II (87.0% vs 88.2%, *P* = 0.788).

In the patients with stages I to III disease, the 5-year survival rate of the HDLN-positive group was significantly lower than that of the HDLN-negative group (59.3% vs 91.2%, *P* < 0.001). There was also a significant difference in 5-year survival rate between patients with stages I to III disease with HDLN metastasis and those with stage IV disease irrespective of HDLN metastasis (59.3% vs 18.8%, *P* = 0.001) (Fig. 1). In the comparison of 5-year survival rate for each stage, the patients with stage III disease with HDLN metastasis showed lower survival rates than those without HDLN metastasis (51.7% vs 66.3%, *P* = 0.001) (Fig. 2).

The univariate and Cox multivariate analysis revealed that HDLN metastasis was an independent prognostic factor (Table 2).

**Table 2:**
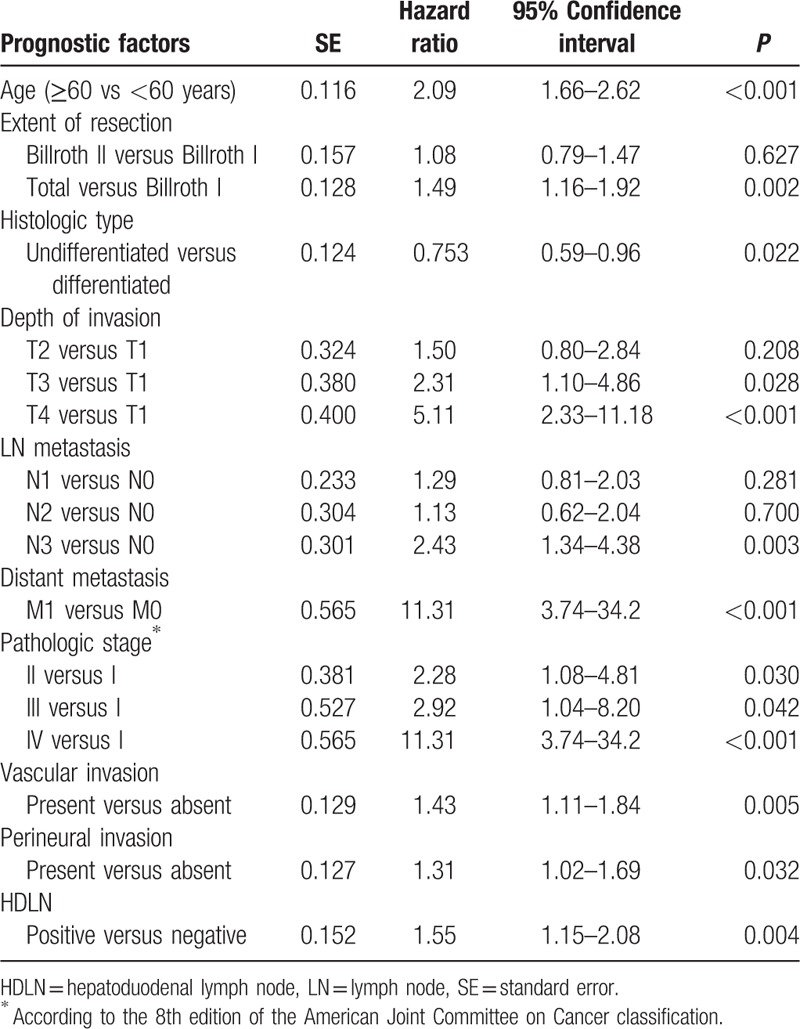
Multivariate logistic regression analysis of prognostic factors of survival.

## 4 Discussion

The present study retrospectively analyzed individual clinical data from 3175 patients who underwent curative resection for gastric cancer and evaluated the impact of HDLN metastasis on the survival outcome of patients. Our result revealed that HDLN has a different prognostic significance from other regional LNs in advanced stage of gastric cancer though its positivity is not considered as distant metastasis.

HDLN metastasis has been one of the controversial issues regarding D2 dissection because it was considered distant metastasis in the previous AJCC classification (7th edition),^[9,10]^ whereas previous studies have shown that No. 12a LN was not different from other regional LNs in terms of prognostic impact and should be treated as such.^[15,16]^

The results of our study correspond with the findings of earlier studies in that the survival rate of patients with HDLN metastasis was better than that of those with distant metastasis. In this regard, it seems appropriate that HDLN should be recognized as one of the regional LNs as classified in the 8th AJCC criteria and HDLN should be removed in D2 dissection. However, for stage III disease, the patients with HDLN metastasis had a worse prognosis than those without HDLN metastasis, and multivariate analysis also revealed that HDLN positivity was an independent prognostic factor. Contrary to the conclusion of previous studies, our results suggest that HDLN metastasis has a different prognostic effect from other regional LNs. The survival outcomes of patients with stage III disease need to be reassessed according to HDLN positivity.

Some differences exist between our study and previous studies. The present study included a larger number of patients with shorter inclusion period and also compared the survival outcome of HDLN-positive and -negative groups according to stage; no stage-by-stage comparison was performed in the earlier studies. As in any other retrospective studies, limitation of the current analysis includes possible selection bias, detection bias. The current study also had some limitations of partial evaluation of HDLN including No. 12a LN only, except for other components in HDLN such as LN along the bile duct (12b) and along the portal vein (12p). We also excluded patients who had neoadjuvant treatment which may influence nodal disease. This exclusion may limit the external validity of our study in other countries where primary surgery for advanced gastric cancer with positive nodes is not the standard of treatment.

In conclusion, HDLN positivity itself seems to be an independent prognostic factor. It seems appropriate that HDLN metastasis in gastric cancer should not be treated as distant metastasis as other previous studies have suggested, and HDLN should be removed during D2 gastrectomy. However, contrary to the conclusion of previous studies, our study revealed that the survival rate of the HDLN-positive group was significantly lower than that of HDLN-negative group in patients with stage III disease. HDLN positivity is associated with worse prognosis in advanced stages, and HDLN should be considered differently from other regional LNs in terms of prognostic significance.
